# The biomass of bloom-forming colonial *Microcystis* affects its response to aeration disturbance

**DOI:** 10.1038/s41598-022-25017-4

**Published:** 2022-12-05

**Authors:** Xiaodong Wang, Xingguo Liu, Boqiang Qin, Xiangming Tang, Xuan Che, Yanqing Ding, Zhaojun Gu

**Affiliations:** 1 Fishery Machinery and Instrument Research Institute, Chinese Academy of Fishery Sciences, 63 Chifeng Rd., Shanghai, 200092 China; 2 State Key Laboratory of Lake Science and Environment, Nanjing Institute of Geography and Limnology, Chinese Academy of Sciences, Nanjing, 210008 China; 3 School of Resources and Geosciences, China University of Mining and Technology, Xuzhou, 221116 Jiangsu China

**Keywords:** Environmental sciences, Limnology

## Abstract

The algal succession in *Microcystis* blooms of varying biomass under continuous aeration was studied in a greenhouse. There were four treatments (control, Low, Medium, and High) with initial chlorophyll *a* (Chl-*a*) of 32.5, 346.8, 1413.7, and 14,250.0 μg L^−1^, respectively. During the experiment, Cyanophyta biomass was the lowest in the Medium treatment (*P* < 0.05), while its Chlorophyta biomass was the highest (*P* < 0.05). Both Chlorophyta and Bacillariophyta biomass were the lowest in the High treatment (*P* < 0.05). Bacillariophyta biomass, particularly the diatom *Nitzschia palea* was the highest in the Low treatment (*P* < 0.05), and *Nitzschia palea* cells were attached to the *Microcystis* colonies. Thus, the algal shift in *Microcystis* blooms under aeration disturbance depends on its initial biomass, and it shift to green algae or/and diatom dominance in the control, Low, Medium treatments. Diatom cells, particularly *N. palea,* grew in an attached form on *Microcystis* colonies in treatment Low, in which the colonies provided media for the adherence. The mechanism of the algal shift with different biomass must be related to the nutrient level, low light and aerobic conditions under aeration disturbance as well as the aeration itself, which destroyed the *Microcystis* colonies’ advantage of floating on the water.

## Introduction

Harmful algal blooms (HABs) are a major environmental problem globally^1^. In addition to harming human beings, livestock, fish and other organisms, HABs can also affect ecosystem structure and function, water supply and irrigation, leisure activities, tourism, and aquaculture^2,3^. There are many kinds of HABs, but the blooms in inland freshwaters are mainly caused by floating cyanobacteria, particularly *Microcystis*^4–6^. Therefore, methods to control *Microcystis* blooms are urgently required.

*Microcystis* blooms consist of many *Microcystis* colonies which can form fine aggregates. These aggregates can be up to several millimeter and float on the water instead of being suspended in the water column when the hydrodynamic force is not strong enough. Extracellular polysaccharides (EPS) in the mucilage sheath supports *Microcystis* colony formation^7^.

Once a large mass of the floating *Microcystis* colonies accumulates, a black bloom, also known as “black spot” or “dead zone” occurs^8^. Algae-induced black blooms, which can produce toxins and trigger both the collapse of lake ecosystems and crises in urbanwater supplies, have become a serious ecological problem in numerous eutrophic shallow lakes. A lot of attention has been paid to the process of black bloom formation, particularly the decomposition of *Microcystis* blooms of different biomass^9,10^. Many factors affect the formation of black blooms, in which the low levels of dissolved oxygen (DO) is very important^8^. Preventing and suppressing the outbreak of black blooms is critical for maintaining the health of lake ecosystems. One important method to control black bloom formation is increasing DO by hydrodynamic force, which has been suggested as an important strategy against the recurrence of a water pollution crisis in Lake Taihu, China.

Additionally, hydrodynamic force can control cyanobacterial blooms. Some studies have found that hydrodynamic disturbance can regulate cyanobacterial blooms, particularly *Microcystis* blooms, to diatom dominance^11–14^. Artificial disturbance reduced the growth of *Microcystis* in Nieuwe Meer Lake in the Netherlands^12^, and the *Microcystis* bloom changed to a diatom and green algae bloom under high-intensity hydrodynamic disturbance^11,13^. Artificial disturbance caused a cyanobacterial bloom to shift to a diatom bloom in Ford lake^14^. Solar powered circulation can effectively control freshwater HABs^15^. This control method is eco-friendly and environmentally sustainable.

A number of factors affect the process of hydrodynamic disturbance in controlling *Microcystis* blooms*.* The abundance of *Microcystis* and the disturbance intensity were key factors affecting the phytoplankton of Hartbeespoort Dam in South Africa^16^. It can be seen that the shift from a *Microcystis* bloom to a diatom bloom under hydrodynamic disturbance is related to the biomass of the *Microcystis* bloom. The biomass of *Microcystis* bloom also affected bacterial community composition in anoxic conditions^10,17,18^. However, few researches on algal composition has been undertaken in aerobic conditions with *Microcystis* blooms of different biomass^16^.

The question then arises, what would happen to varying biomass of bloomed *Microcystis*, if enough oxygen was provided through hydrodynamic force, particularly aeration? If the DO was high enough to prevent the formation of a black bloom, then what changes would occur in the *Microcystis* colonies? Will any other algae, such as green algae and diatoms appear during the aeration disturbance? If so, which species? In order to answer these important questions, an experiment on the effect of aeration disturbance on *Microcystis* blooms of varying biomass was carried out in a greenhouse.

## Results

### Light in the greenhouse

The glass of the greenhouse shaded the sunshine, and so the light levels inside the greenhouse were lower than the natural light levels outdoors. The shading rate in the greenhouse was about 50% (Table 1).

**Table 1:** The PAR both in the greenhouse and the outdoor on a sunny day, and the calculated shading rate of the greenhouse.

Time	PAR	In the greenhouse	Outdoors	Shading rate of the greenhouse (%)
09:00	Quantum/(μmol m^−2^ s^−1^)	865.73 ± 2.48	1773.97 ± 6.67	51.2
	Energy/(W m^−2^)	166.65 ± 2.01	335.66 ± 0.50	50.4
10:00	Quantum/(μmol m^−2^ s^−1^)	962.45 ± 1.63	1929.30 ± 4.54	50.1
	Energy/(W m^−2^)	197.07 ± 0.99	387.15 ± 1.11	49.1

### Water quality, Chl-*a* and total wet weight

**Figure 1:**
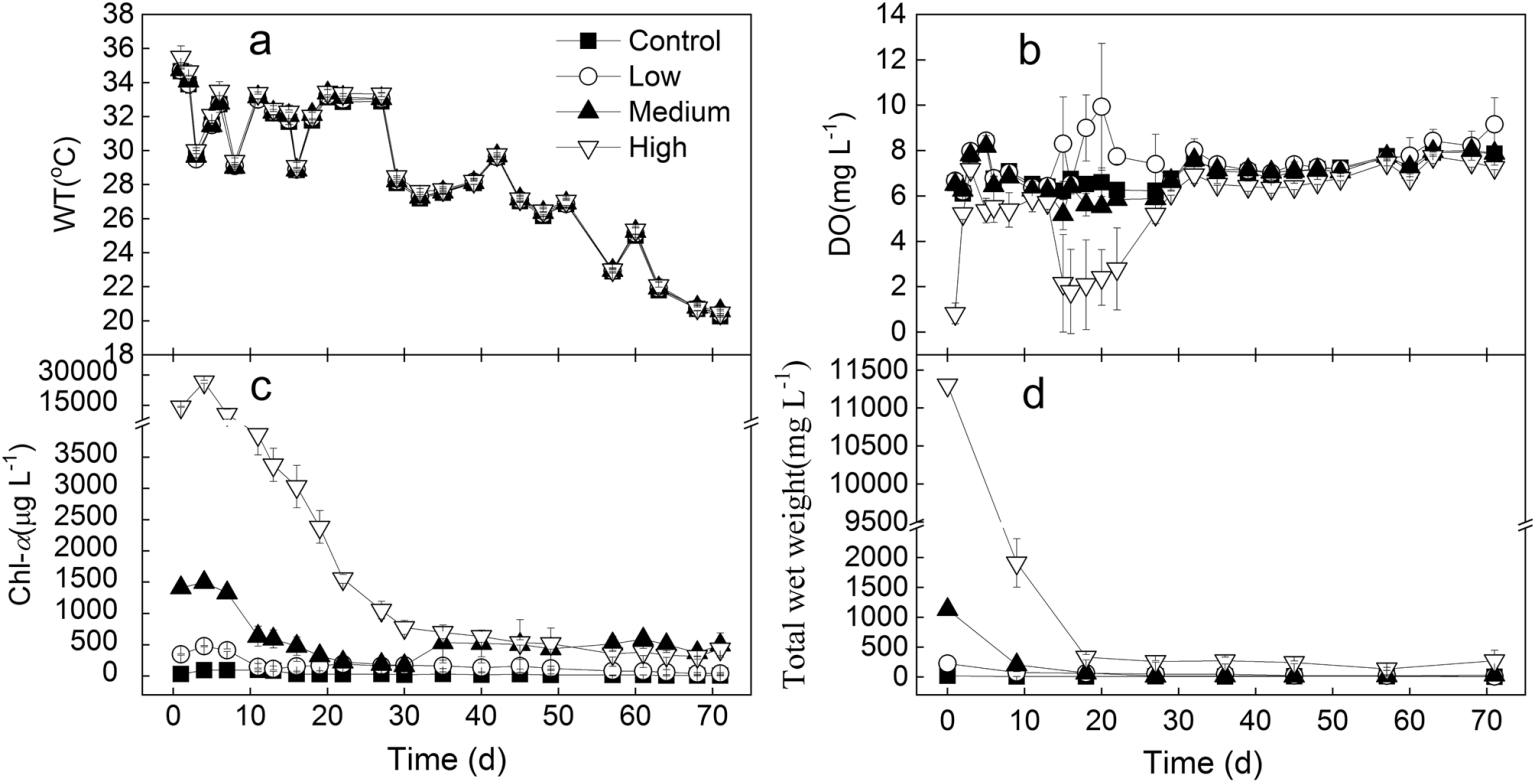
The changes in WT (**a**), DO (**b**), Chl-*a* (**c**) and algal wet weight (**d**) in the four treatment.

The changes in WT, DO, Chl-*a* and total wet weight of the treatments are shown in Fig. 1. During the experiment, the DO in all the treatment was mainly > 5 mg L^−1^, except a few times in treatment High. The ANOVA results showed that DO in the High treatment was significantly lower than the other three treatments (*P* < 0.05), and DO in the Low treatment was also higher than both the control and the Medium treatments (*P* < 0.05). The ANOVA results of both Chl-*a* and total wet weight showed that there were significant differences among the four treatments, and the rank order from highest to lowest was High > Medium > Low > control (*P* < 0.05) (Fig. 1).

### Phytoplankton community structure dynamics

Although the number of phytoplankton species recorded at the start of the experiment was five, a total of 33 phytoplankton species were observed over the entire duration of the experiment. During the experiment, the number of phytoplankton species increased to 21, 27, 18, and 9 in the control, Low, Medium, and High treatment groups, respectively. These species belonged to four major groups: Cyanophyta, Chlorophyta, Bacillariophyta, and Cryptophyta. Cyanophyta and Chlorophyta were the dominant groups throughout the experiment.

**Figure 2:**
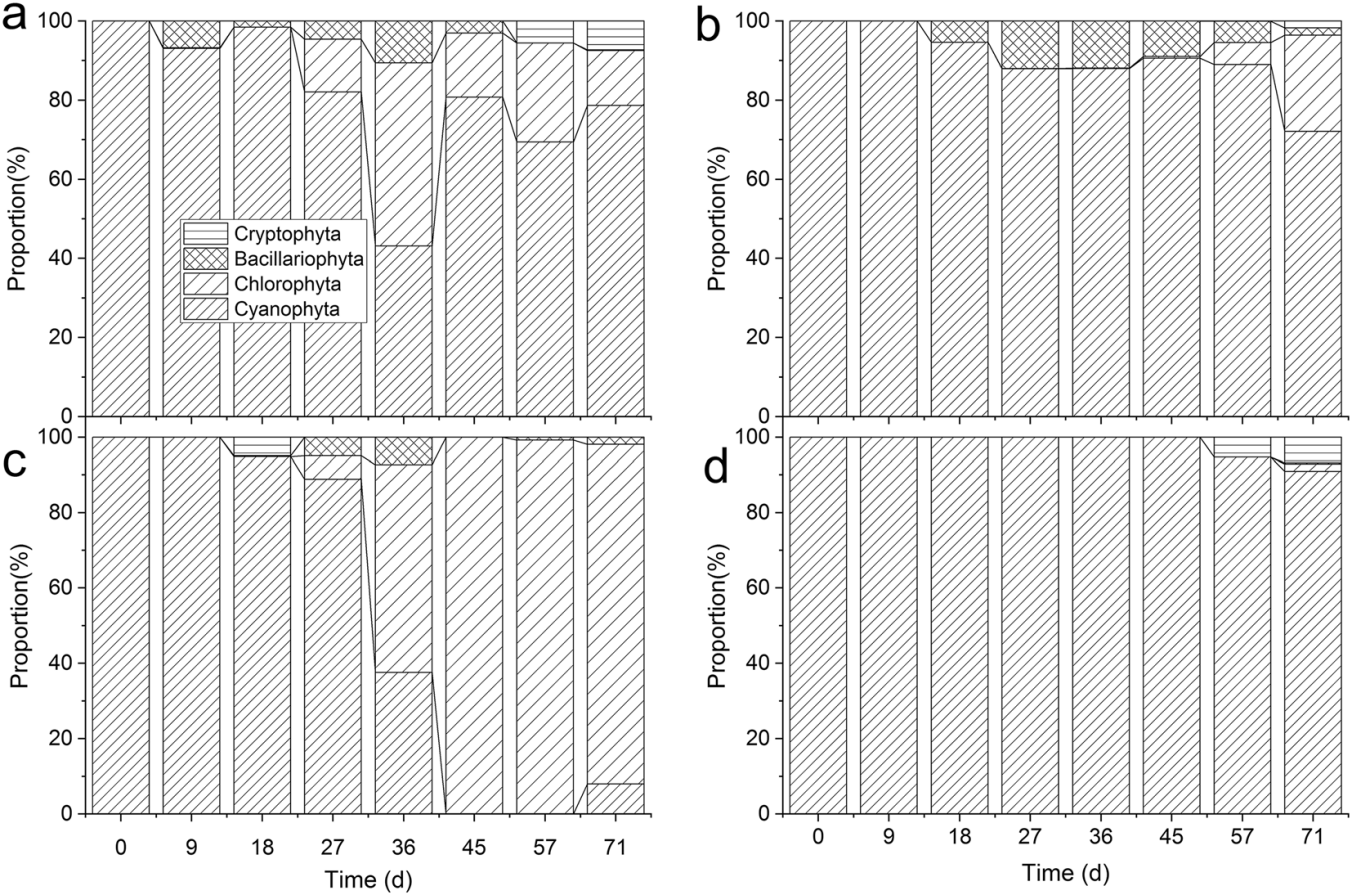
The proprotional contribution of Cyanophyta, Bacillariophyta, Chlorophyta, and Cryptophyta to the total biomass in the control (**a**), treatments Low (**b**), Medium (**c**) and High (**d**) during the experiments.

Figure 2 shows that the algal changes depended on the initial biomass of the *Microcystis* bloom. The algal community dynamically changed during the experiment, and Cyanophyta was always dominant in the control, Low, and High treatments. From about day 27 on, Chlorophyta showed with a certain dominance in the control. Bacillariophyta appeared in treatment Low, and it accounted for about 10% from day 27 to 45. While Chlorophyta became dominant in the Medium treatment from day 36 on.

The ANOVA results for the biomass of different groups showed that the Cyanophyta biomass in the Medium treatment was lowest (*P* < 0.05), and the Chlorophyta biomass in the Medium treatment was highest (*P* < 0.05), while both the Chlorophyta and Bacillariophyta biomass was lowest in the High treatment (*P* < 0.05). The Bacillariophyta biomass was highest in the Low treatment (*P* < 0.05). These showed the algal community changed according to the initial biomass of the *Microcystis* bloom under aeration disturbance. When the concentration of Chl-*a* in the *Microcystis* bloom ranged from 32.52 to 1413.70 μg L^−1^, the algal community diversified, reducing *Microcystis* dominance.

**Figure 3:**
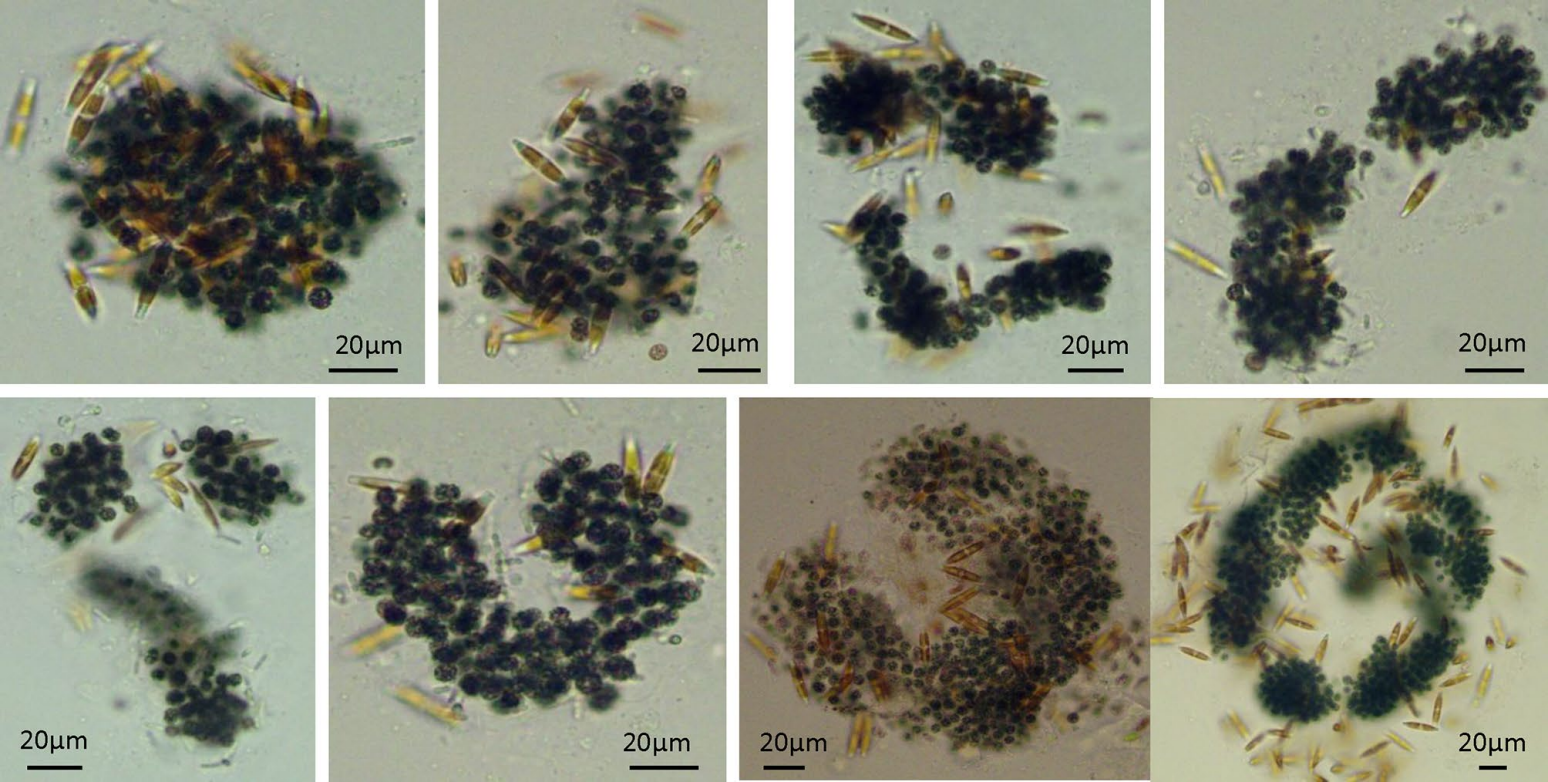
Pictures of the *Nitzschia palea,* a diatom attached to the *Microcystis* colonies in treatment Low on day 20.

Table 2 shows that *Microcystis* was always dominant in the control, and some species of Chlorophyta, Diatom, and Cryptophylum appeared during the experiment. Similarly, in the Low treatment, *Microcystis* was always dominant, and some species of Chlorophyta and Diatom appeared, particularly, *Nitzschia palea*, a diatom which became dominant from day 18 to 57 (Table 3). Some images were taken of this treatment on day 20 (Fig. 3), which showed that the diatom cells were attached to the *Microcystis* colonies. There were few diatoms growing as free-living forms. In the Medium treatment, *Microcystis* was not dominant from about day 45 on, but five Chlorophyta genera became dominant (Table 4). In the High treatment, *Microcystis* was dominant all the time with some diatom and Chlorophyta dominance towards the end of the experiment (Table 5).

**Table 2:** Algal changes in the five dominant genera in the control (wet weight, mg L^−1^).

Phylum	Genera or species	Day 0	Day 9	Day 18	Day 27	Day 36	Day 45	Day 57	Day71
Cynaobacteria	*Microcystis* spp.	21.73	6.38 ± 1.18	7.29 ± 1.44	6.39 ± 0.78	4.14 ± 1.40	10.90 ± 2.65	8.67 ± 2.22	3.94 ± 6.83
	*Anabeana* sp.	0.43	0.29 ± 0.32	0.74 ± 0.73	2.80 ± 0.53	0	0	0	0
	*Planktothricoides*	0.45	0.14 ± 0.16	0	0	0	0	0	0
	*Planktothrix* sp.	0	0	0.03 ± 0.06	0	0	0	0	0
Chlorophyta	*Micractinium* sp.	0	0	0	–	2.14 ± 2.00	0.70 ± 0.94	0	0
	*Coelastrum* sp.	0	0	0	0.66 ± 0.21	0	0.06 ± 0.11	0	0
	*Oocystis* sp.	0	0	0	0	0	–	1.72 ± 1.36	0.48 ± 0.83
	*Chlorella* sp.	0	0	0	0	0	0	0	0.11 ± 0.18
	*Scenedesmus* spp.	0	0.01 ± 0.02	0	0.41 ± 0.16	2.28 ± 1.96	1.05 ± 0.90	1.41 ± 0.74	0.12 ± 0.17
Diatom	*Pediastrum* sp.	0	0	0	–	0.01 ± 0.01	–	0	0
	*Nitzschia* sp.	0	0.50 ± 0.53	0.12 ± 0.22	–	0	0	0	0
	*Coscinodiscus* sp.	0	0	0	0.28 ± 0.37	1.01 ± 1.02	0.41 ± 0.48	0	0
Cryptophylum	*Cryptomonas* sp.	0	0	0	0	0	0	0.70 ± 0.30	0.36 ± 0.48

**Table 3:** Algal changes in the five dominant genera in treatment Low (wet weight, mg L^−1^).

Phylum	Genera or species	Day 0	Day 9	Day 18	Day 27	Day 36	Day 45	Day 57	Day71
Cynaobacteria	*Microcystis* spp.	217.29	71.40 ± 10.22	50.65 ± 12.14	30.65 ± 8.98	39.45 ± 11.35	17.64 ± 12.20	19.52 ± 14.65	3.52 ± 1.34
	*Planktothricoides* sp.	4.51	1.42 ± 1.07	5.06 ± 1.37	9.40 ± 4.18	0.09 ± 0.16	0	0	0
	*Anabeana* sp.	4.27	4.83 ± 1.46	0	0	0	0	0.15 ± 0.26	0.69 ± 1.07
	*Planktothrix* sp.	0	0	2.90 ± 3.66	5.53 ± 1.63	2.54 ± 2.46	3.33 ± 1.73	0	0
	Unknown filaments	0	0	0.10 ± 0.17	0	0	0	0	1.51 ± 2.61
Diatom	*Nitzschia* sp.	0	0	3.33 ± 2.65	6.18 ± 2.78	5.70 ± 4.22	2.07 ± 0.77	1.19 ± 0.46	**–**
	*Fragilaria* sp.	0	0	0	0.04 ± 0.08	0	0	0	0
Chlorophyta	*Scenedesmus* spp.	0	0	0	**–**	0.05 ± 0.08	0.11 ± 0.14	1.10 ± 1.58	0.64 ± 0.22
	*Pediastrum* sp.	0	0	0	0	0	0	0.10 ± 0.18	0.59 ± 1.03
	Unknown thin rod cells	0	0	0	0	0	0.10 ± 0.17	0	0

**Table 4:** Algal changes in the five dominant genera in treatment Medium (wet weight, mg L^−1^).

Phylum	Genera or species	Day 0	Day 9	Day 18	Day 27	Day 36	Day 45	Day 57	Day71
Cynaobacteria	*Microcystis* spp.	1086.42	196.00 ± 18.18	51.72 ± 10.84	7.18 ± 0.97	4.85 ± 2.27	0	0	0
	*Planktothricoides* sp.	22.50	8.51 ± 3.30	10.33 ± 1.71	4.48 ± 1.13	2.46 ± 1.50	0	0	0
	*Anabeana* sp.	21.31	0	0.17 ± 0.26	0	0	0	0	0
	*Planktothrix* sp.	0	0	2.12 ± 1.27	1.44 ± 1.01	–	0	0	0
	*Dactylococcopsis* sp.	0	0	0	0	0	0	0	2.10 ± 2.15
Diatom	*Nitzschia* sp.	0	0	–	0.73 ± 0.79	–	0	0.17 ± 0.29	–
Cryptophylum	C*ryptomonas* sp.	0	0	3.32 ± 2.53	0	0	0	0	0
Chlorophyta	*Ankistrodesmus* spp.	0	0	0	–	4.45 ± 2.00	9.95 ± 7.30	7.23 ± 3.79	5.66 ± 5.52
	*Scenedesmus* spp.	0	0	0	–	3.10 ± 2.78	2.94 ± 2.12	1.61 ± 1.08	11.96 ± 10.74
	*Chlorella* sp.	0	0	0	0.43 ± 0.47	3.02 ± 0.95	4.23 ± 2.88	11.79 ± 6.55	
	*Micractinium* sp.	0	0	0	0	–	0	0	5.58 ± 9.45
	*Chl-amydomonas* sp.	0	0	0	0	0	0	0	0.68 ± 0.64

**Table 5:** Algal changes in the five dominant genera in treatment High (wet weight, mg L^−1^).

Phylum	Genera or species	Day 0	Day 9	Day 18	Day 27	Day 36	Day 45	Day 57	Day71
Cynaobacteria	*Microcystis* spp.	10,864.25	1902.19 ± 406.14	331.23 ± 45.09	260.32 ± 10.98	273.33 ± 54.29	244.29 ± 24.72	136.89 ± 25.11	154.83 ± 62.62
	*Planktothricoides* sp.	225.00	5.53 ± 0.55	0	0	0	0	0	0
	*Anabeana* sp.	213.12	2.72 ± 0.30	0	0	1.28 ± 1.78	0	0	0
Diatom	*Nitzschia* sp.	0	0	0	0	0	0	0	0.53 ± 0.46
Chlorophyta	*Micractinium*	0	0	0	0	0	0	0	1.11 ± 1.91
	*Dictyosphaerium* sp*.*	0	0	0	0	0	0	0	2.28 ± 3.96

ANOVA results showed that the biomass of the diatom *N. palea* was highest in the Low treatment, and it was significantly higher than the other three treatments (*P* < 0.05), while that in the High treatment was also significantly lower than treatment Medium (*P* < 0.05). ANOVA results on the total biomass (wet weight) showed its rank order was High > Medium > Low > control (*P* < 0.05).

**Figure 4:**
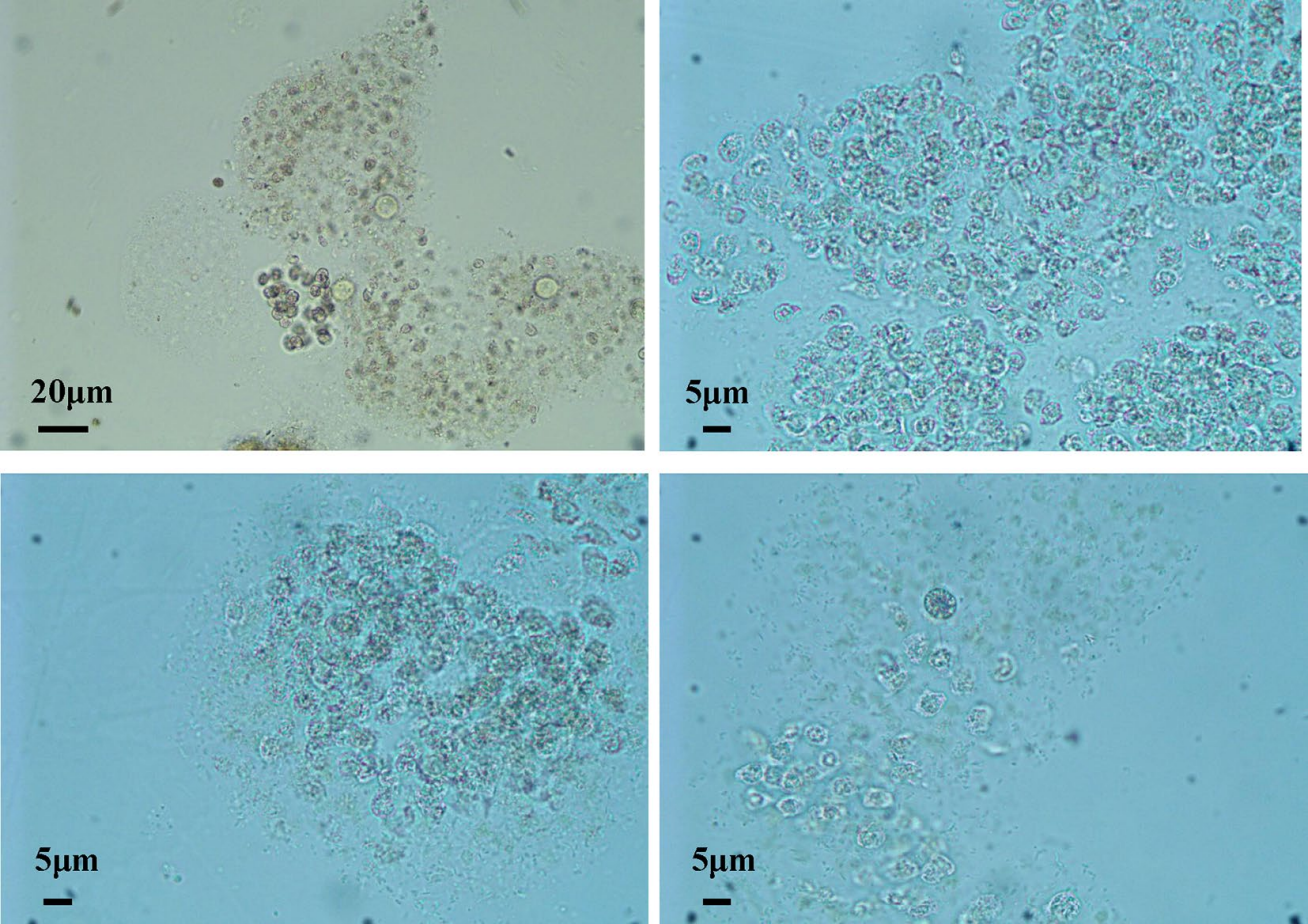
Pictures of the *Microcystis* colonies during the decomposition in treatment High on day 30.

A species of diatom *N. palea* was recorded during the experiment. These diatom cells were found to be attached to the mucilaginous sheath of *Microcystis* colonies instead of free-living. Several to a few hundreds of these diatoms could be present in each single *Microcystis* colony (Fig. 3). No colony-attached growth of green algae was found in the *Microcystis* colonies in all the treatments. On the other hand, the decomposition of the *Microcystis* colonies can be found in treatment High (Fig. 4), in which neither diatoms nor green algae were attached.

## Discussion

The experiment results showed that the algal community response depended on the initial *Microcystis* biomass (Figs. 1, 2, 3, Tables 2, 3, 4, 5), and a number of green algae and a few diatom species grew during the experiment. These findings were consistent with the scientific hypothesis that green algae and diatoms can succeed from the initial bloom-forming colonial *Microcystis* under aeration disturbance. In particular, the diatom, *N. palea* showed a certain dominance, adhering to *Microcystis* colonies in the Low treatment (Fig. 3), which is very interesting.

The treatments were all provided with aeration disturbance, while many studies have found that hydrodynamic turbulence/mixing can change phytoplankton community composition^13,19^. Turbulence can select for particular life-forms^20^. Changes in the intensity and extent of turbulence in natural water bodies, such as those driven by climate change, may induce species replacements from cyanobacteria in lakes/rivers towards diatom/green algae-dominated communities^13^. Experiments carried out with *Microcystis* blooms from Lake Taihu, China showed that mixing induced shifts in the phytoplankton community, favoring the dominance of diatoms and green algae^21^.

Hydrodynamic disturbance was used to control *Microcystis* blooms. The goal of ecological approaches to *Microcystis* bloom control is to enable beneficial phytoplankton to outcompete cyanobacteria. Deep mixing hampered the growth of the cyanobacteria *Microcystis* and *Anabaena*, while diatoms (*Asterionella*, *Fragilaria*, and *Staurastrum*) were favoured by deep mixing^22^, which caused a shift from cyanobacteria to green algae and diatoms. Hydrodynamic disturbance can reduce the growth of *Microcystis*, and even make the shift from *Microcystis* blooms into diatom and green algae blooms^11–13^. Strong disturbance can promote the succession of phytoplankton dominant species from *Microcystis* to diatoms and green algae in deep-water lakes^23^.

Artificial aeration, a type of hydrodynamic disturbance, is commonly used to control water quality and phytoplankton communities in reservoirs^24,25^. Artificial aeration in a small tropical reservoir changed the make-up of the phytoplankton community, replacing dominant bloom-forming cyanobacteria with diatoms, without reducing the total algal standing crop^24^. Aeration destratified the water column of the reservoir, Lake Dalbang (South Korea), and prevented cyanobacteria blooms^25^. A common beneficial result of destratification caused by artificial mixing in deep lakes was the replacement of cyanobacteria with diatoms^26^.

Artificial mixing as a solution to prevent harmful cyanobacterial blooms was reviewed^19^. The shift from cyanobacteria to green algae and diatoms was affected by many factors, such as buoyancy regulation, temperature, oxygen, nutrients, and light, and it was considered that the shift mechanism was well understood in an artificially mixed system^19^. Under artificial turbulence, *Microcystis* colonies lose their advantage of buoyancy to float on the water^16^. Green algae and diatoms which are negatively buoyant, profited from the mixed conditions with fluctuating irradiance^13,16,19^.

Then the effect of frequency and intensity of disturbance on the algal shift was considered^27–29^. The intensity of disturbance can affect the growth of *Microcystis* by changing the absorption and utilization of P by *Microcystis*^27^. The disturbance frequency can affect the algal shift, including that continuous hydrodynamic mixing weakens the dominance of *Microcystis*^28^ and intermittent disturbance benefits colony size, biomass and dominance of *Microcystis* in Lake Taihu under simulated field conditions^29^.

However, the effect from the biomass of bloom-forming colonial *Microcystis* was not reviewed by Visser et al.^19^, as few researches payed attention to effects from algal biomass^16^. Research on the algae of Hartbeespoort Dam in South Africa^16^ showed that the abundance of *Microcystis* was an important factor affecting the algal shift under hydrodynamic disturbance. When *Microcystis* was rare, the green algae *Carteria cordiformis, Dictyosphaerium pulchellum, Pandorina* spp., and *Scenedesmus linearis* tended to increase across a broad spectrum of temperature and nutrient conditions^16^. On the contrary, the diatoms *Cyclotella meneghiniana* and *Melosira* (syn. *Aulacoseira*) *granulata* and the cryptophytes *Chroomonas* sp. and *Cryptomonas* sp. occurred more frequently with high *Microcystis* abundances^16^.

Actually, there have been some failures to suppress freshwater HABs in ponds with artificial mixing and solar powered circulation^15^, the cause of which is unknown^15^. Moreover, hydrodynamic disturbance may promote *Microcystis* blooms under some conditions^30^. It is generally believed that wind and wave disturbance contributes to the formation of *Microcystis* blooms in the shallow eutrophic Taihu Lake, China, and the main mechanism was that the wind and wave disturbance led to sediment resuspension and the utilization of nutrients by algae^31–33^. Thus, the influence of hydrodynamic disturbance on *Microcystis* blooms has not been fully studied^34^, which increases the complexity of hydrodynamic disturbance in regulating *Microcystis* blooms.

Temperature is also an important factor affecting the algal shift. Eutrophication and warming are widely recognized as the main drivers of cyanobacterial blooms^35–37^. One of the mechanisms that cyanobacteria outcompete their eukaryote competitors at elevated temperatures is the direct warming effect on growth rates^35,38,39^. During this experiment, the water temperature gradually reduced from about 34 to 20 °C (Fig. 1). Then it seems that the appearance of diatoms in treatment Low and Medium was related to the gradually decreased temperature, however, the peak of diatom cells was from about day 27 to 36 in all the control, treatments Low and Medium, when the water temperature was from about 34 to 26 °C. Then the diatoms didn’t maintain the peak density at the later stage, and the water temperature was lower. On the other hand, green algae dominated in treatment Medium from day 36 on. Thus the gradually reduced water temperature from about 26 to 20 °C at the later stage was not the reason for the occurrence of diatoms in the control, and treatments Low and Medium.

Light and nutrients are also important factors affecting the algal shift. Diatoms are generally believed to have relatively low light requirements, and the decline of spring diatom blooms in summer is due to strong illumination, which reduces the reproduction rate of diatom populations and accelerates settlement^40^. The *Microcystis* bloom was comprised of *Microcystis* colonies, which was in fine aggregates. They cannot stay floating on the water and were physically mixed into the water column under aeration disturbance, producing “self-shading” and fluctuating light conditions.

Although the light intensity in the tanks was not measured in this experiment, it can be deduced that the higher the biomass, the lower the light intensity in the tanks. Moreover, Chl-*a* concentration in all treatments decreased during the aeration process, indicating that some *Microcystis* colonies must have decomposed to supply dissolved nutrients for algal growth. And Fig. 4 showed the decomposition of *Microcystis* colonies in treatment High. Similarly, algal decomposition can release some SRP and NH_4_-N to support other algae^41^. And the decomposition of *Microcystis* blooms has been reported to produce nutrients^10,17,18^. Therefore, light intensity and nutrients must differ with varying *Microcystis* bloom biomass. Evenmore, this experiment was carried out in a greenhouse with a shading rate of about 50% (Table 1). So the low and fluctuating light, as well as nutrients, must have affected the algal response under aeration disturbance.

After studying the algal blooms and algal shift under hydrodynamic disturbance, a theory of light competition was established^13^, which means the changes brought about by mixing affects competition for light between buoyant and sinking phytoplankton species in eutrophic conditions. It is believed that in highly eutrophic waters, when algae are fully mixed, that light, not nutrients, will be the limiting factor^13,30^. This light competition theory emphasizes the important role of low light conditions in the succession of algal communities. Moreover, Robarts and Zohary^42^ found that the size of the *Microcystis* colony moderated the effect of chlorophyll concentration on light attenuation. And attenuation of light even influences the size of *Microcystis* colonies^43^.

According to the theory of light competition^13^, diatoms should be dominant in all the Low, Medium, and High treatments, as nutrient levels were high and light density was low. However, that was not the case in the current study, where green algae dominated in treatment Medium, and *Microcystis* remained dominant in treatment High (Tables 2, 3, 4, 5). These results showed that only a certain range of low light resulting from the self-shading was enough for diatom growth. Then the role of low light and high nutrient for the algal shift may be not equally important with varying *Microcystis* biomass. The role of low light may be more effective than high nutrient for the shift to more diatom cells in treatment Low.

And researches on the nutrients level on algal shift found that green algae dominance instead of diatom dominance tended to occur with nutrients enrichment^44–46^, which was similar to the algal shift in treatment Medium. The dominant algae shift to green algae in treatment Medium, which was different from the algal shift in the other three treatments. The role of high nutrient may be more effective than low light for the shift to green algae dominance in treatment Medium. However, the High treatment was not the most propensity for the changing of phytoplanktonic community composition. The most important reason may be that the density of *Microcystis* colonies was too high. The *Microcystis* colonies gradually decomposed and bacteria instead of algae may be dominant (Fig. 4).

In this experiment, the most interesting phenomenon was that the *N. palea* cells were attached to the mucilaginous sheath of *Microcystis* colonies (Fig. 3), which was different from the free-living growth. *N. palea* is regarded as an indicator of eutrophication and commonly occurs in polluted rivers in urbanized areas^47^. Similarly, the filamentous cyanobacterium *Pseudoanabaena* sp. can grow in attachment to *Microcystis* colonies, and diatoms co-existed with *Microcystis* in African freshwater lakes^48^.

The same aeration disturbance was provided to the four treatments in this experiment, which improved the DO level, mainly maintaining at > 5 mg L^−1^ (Fig. 1). The initial Chl-*a* concentration in the Medium treatment was close to the level of 2000 μg L^−1^ in a study^10^, and it reached the lowest value in about 30 days, which was about 250 μg L^−1^ (Fig. 1). However, under incubation in the dark with no hydrodynamic disturbance, the Chl-*a* concentration decreased from 2000 to 5 μg L^−1^ in 14 days, decreasing particularly sharply over the first 4 days^10^. The DO level decreased to 1.15 (± 0.15) mg L^−1^ on day 6^10^. In comparison to the decomposition of *Microcystis* blooms in anoxic or anaerobic conditions^10^, the decomposition in the current study was much slower. The aerobic conditions caused by the aeration disturbance slowed down the decomposition of *Microcystis* colonies in comparison with anoxic or anaerobic conditions.

The diatom cells were attached to the *Microcystis* colonies in this experiment (Fig. 3). The aggregation of cells in *Microcystis* colonies is due to a mucilaginous sheath, which mainly consists of EPS^7^. Then it is possible that the *Microcystis* colonies, particularly the EPS, decomposed much slower in aerobic conditions than in anoxic or anaerobic conditions, and the mucilage sheath that was made of EPS provided a physical media for the attached growth of diatoms under aeration disturbance. Therefore, the diatom response is related to the aeration mixing itself and the aerobic conditions in this experiment. Based on the above analysis, the EPS of *Microcystis* colonies provided a physical media for the attachment growth of diatoms under aeration disturbance.

In conclusion, our results reveal that the biomass of bloom-forming colonial *Microcystis* affects its response to aeration disturbance. When the initial *Microcystis* bloom was 32.5, 346.8, or 1413.7 μg L^−1^ Chl-*a*, the algal community changed to green algae or/and diatom dominance. When the initial Chl-*a* of the *Microcystis* bloom was 346.8 μg L^−1^, diatoms, particularly *N. palea,* grew in an attached form on *Microcystis* colonies from about days 10 to 57. Cyanobacteria remained dominant when the initial Chl-*a* of the *Microcystis* bloom was 14,250 μg L^−1^. The mechanism of the algal shift with different biomass must be related to the nutrient level, low light and aerobic conditions under aeration disturbance as well as the aeration itself, which destroyed the *Microcystis* colonies’ advantage of floating on the water.

## Methods

### Experimental design

On the 9th of August, 2018, a *Microcystis* bloom was obtained from an aquaculture pond containing mainly *Megalobrama amblycephala*, located in the Songjiang District of Shanghai, China. The bloom was dominated by *Microcystis* spp., particularly *M. aeruginosa*. The bloom was condensed into a thick slurry with a 200-mesh nylon screen. A large amount of tap water was used to wash the thick bloom slurry through the 200-mesh nylon screen, to dilute the dissolved nitrogen and phosphorus nutrients in the *Microcystis* bloom as close to the level of tap water as possible. The concentrations of total nitrogen (TN) and total phosphorus (TP) in the tap water were 1.495 and 0.045 mg L^−1^, respectively.

After washing with tap water, the thick bloom slurry was transferred to transparent borosilicate 10 L glass tanks (23 cm diameter, 35 cm high) in a greenhouse. Tap water was then used to dilute the thick bloom slurry to obtain different biomass of *Microcystis* (Table 6). The total volume of the culture was 10 L of each tank after the dilution. The experiment lasted 71 days.

**Table 6:** The TN, TP and Chl-a contents of each treatment at the start.

Biomass level	TN (mg L^−1^)	TP (mg L^−1^)	Chl-*a* (μg L^−1^)
Control	2.581 ± 0.157	0.087 ± 0.006	32.52 ± 3.16
Low	11.781 ± 0.619	0.886 ± 0.085	346.81 ± 10.19
Medium	61.200 ± 0.799	5.250 ± 0.421	1413.70 ± 37.83
High	598.517 ± 7.989	52.092 ± 4.210	14,250.00 ± 308.67

Chlorophyll-*a* (Chl-*a*) concentration was chosen to reflect the biomass of the *Microcystis* bloom. There were four treatments, with varying levels of *Microcystis* biomass, each with three replicates (Table 6). The concentration of Chl-*a* in the control was 32.52 μg L^−1^, which was similar to the common level of *Microcystis* blooms in Lake Taihu, China^49^. The concentration of Chl-*a* in the other three treatments from Low to High was approximately 10, 50, and 500 times that of the control, respectively. The initial TN, TP, and Chl-*a* concentration of the treatments is also shown in Table 6.

Each tank was continuously aerated with a bubble stone. The aeration intensity was approximately 0.4 m^3^ h^−1^ from day 0 to 14, and day 30 to 71, and approximately 0.2 m^3^ h^−1^ from day 15 to 29. Aeration maintains the suspension of *Microcystis* colonies as much as possible, and prevents water splashing. No sediment was provided.

### Shading rate in the greenhouse and its measurement

In order to determine the shading rate in the greenhouse, the photosynthetically available radiation (PAR) was measured inside and outside the greenhouse at about 09:00 h and 10:00 h, on a sunny day. PAR quantum (unit: μMol m^−2^ s^−1^) and PAR energy (unit: W m^−2^) was measured by a Spectrosense2 meter associated with a four-channel sensor (Skye Instruments, UK). The wavelength of the PAR analysis was 400–700 nm. It was measured three times in three minutes, and the average values were calculated to obtain the light transmittance ratio from the light intensity in the greenhouse and outdoors. The shading rate in the greenhouse was calculated as (%): shading rate (%) = 100 − Transmittance (%).

### Water quality and Chl-*a* measurements

During the experiment, water temperature (WT) and DO were measured every 2–4 days. A YSI multi-parameter water quality monitor meter (YSI professional plus, Yellow Spring Instruments, USA) was used to measure WT and DO in situ at about 14:00 h. Measurements of TN and TP were simultaneously digested according to the methods of Gross and Boyd^50^. Chl-*a* was determined by a PHYTO-PAM (Waltz, Effeltrich, Germany) chlorophyll fluorescence meter, and the software was phytowin2.13.

### Algae identification and counting

Samples for algae identification were collected eight times during the experiment, with a sampling frequency of once every nine days in the first 45 days. To determine algal density, 50-mL or 100-mL water samples were preserved with 1% Lugol’s solution and stored in darkness until required for analysis. If algal density was too low to count, the sample was concentrated after settling. For enumeration, two replicate aliquots were enclosed in 0.1-mL plankton counting chambers that were modified from the Palmer and Maloney design^51^. Most cells were observed at 400× magnification by light microscopy with an Olympus CX31 (Olympus, Japan), while large algal cells were observed at 100× magnification. They were mainly identified to the species level as referenced by morphologies^52^. For the enumeration of cells in *Microcystis* colonies, subsamples were heated to 60 °C for 2 to 4 h to disintegrate the colonies.

Algal volumes were calculated based on cell density and cell size measurements. Calculation of cell volumes was according to their shape, and measurements of length, height, and diameter were obtained to calculate the volume. Cells with irregular shapes were decomposed to some approximately regular geometry, and then the sum value was calculated as the total volume. At least 40 algal units were measured to obtain the average cell volume for each genera or species. The conversion to wet weight biomass assumed that 1 mm^3^ of volume was equivalent to 1 mg of wet weight biomass^45^.

### Data analysis

Data comparison among the treatments was conducted with SPSS 16.0 software for Windows (Statistical Product and Service Solutions, IBM, New York, USA) using two factor analysis of variance (ANOVA) (biomass × time) in the general linear model. To improve the homogeneity of variances, the data of each treatment were square root transformed before the comparison^53^. All data are presented as the mean ± SD. Differences were considered significant if *P* < 0.05.

## Data Availability

The data that support the findings of this study are available from the corresponding author and XW, upon reasonable request.
